# Bilateral exchange in synthetic biology: experiences, challenges, and opportunities for training and research

**DOI:** 10.1093/synbio/ysag012

**Published:** 2026-10-24

**Authors:** María del Carmen Sánchez Olmos, Martha Lorena Medina, Adán Andrés Ramírez Rojas, Nohora Angélica Millán Aldana, Paula Manuela Siaucho Unriza, Nicolas Oscar Londoño Sanchez, Valentina Flórez Téllez, Victor Hugo Rua Lopez, Patricia del Portillo, Maryam Chaib De Mares, María Francisca Villegas-Torres, Daniel Schindler, María Mercedes Zambrano

**Affiliations:** Max Planck Institute for Terrestrial Microbiology, Karl-von-Frisch-Straße 10, 35043 Marburg, Germany; Center for Molecular Biology of Heidelberg University (ZMBH), Heidelberg University, INF 329, 69120 Heidelberg, Germany; Molecular Genetics, Corpogen Research Center, Carrera 4 No. 20-41, Bogotá D.C. 110311, Colombia; Universidad Central, Carrera 5 No. 21-38, Bogotá D.C.110311, Colombia; Max Planck Institute for Terrestrial Microbiology, Karl-von-Frisch-Straße 10, 35043 Marburg, Germany; Max Planck Institute for Terrestrial Microbiology, Karl-von-Frisch-Straße 10, 35043 Marburg, Germany; Grupo de Biología Molecular Teórica y Evolutiva, Departamento de Biología, Facultad de Ciencias, Universidad Nacional de Colombia, Bogotá 111321, Colombia; Center for Molecular Biology of Heidelberg University (ZMBH), Heidelberg University, INF 329, 69120 Heidelberg, Germany; Max Planck Institute for Terrestrial Microbiology, Karl-von-Frisch-Straße 10, 35043 Marburg, Germany; Centro de Investigaciones Microbiológicas (CIMIC), Department of Biological Sciences, Faculty of Sciences, Universidad de los Andes, Bogotá D.C. 111711, Colombia; Center for Molecular Biology of Heidelberg University (ZMBH), Heidelberg University, INF 329, 69120 Heidelberg, Germany; Grupo de Biología Molecular Teórica y Evolutiva, Departamento de Biología, Facultad de Ciencias, Universidad Nacional de Colombia, Bogotá 111321, Colombia; Centro de Investigaciones Microbiológicas (CIMIC), Department of Biological Sciences, Faculty of Sciences, Universidad de los Andes, Bogotá D.C. 111711, Colombia; Grupo de Diseño de Productos y Procesos (GDPP), Department of Chemical and Food Engineering, Faculty of Engineering, Universidad de los Andes, Bogotá D.C.111711, Colombia; Molecular Genetics, Corpogen Research Center, Carrera 4 No. 20-41, Bogotá D.C. 110311, Colombia; Universidad Central, Carrera 5 No. 21-38, Bogotá D.C.110311, Colombia; Grupo de Biología Molecular Teórica y Evolutiva, Departamento de Biología, Facultad de Ciencias, Universidad Nacional de Colombia, Bogotá 111321, Colombia; Centro de Investigaciones Microbiológicas (CIMIC), Department of Biological Sciences, Faculty of Sciences, Universidad de los Andes, Bogotá D.C. 111711, Colombia; Max Planck Institute for Terrestrial Microbiology, Karl-von-Frisch-Straße 10, 35043 Marburg, Germany; Center for Molecular Biology of Heidelberg University (ZMBH), Heidelberg University, INF 329, 69120 Heidelberg, Germany; Center for Synthetic Microbiology, Philipps-University Marburg, Karl-von-Frisch-Straße 14, 35032 Marburg, Germany; Molecular Genetics, Corpogen Research Center, Carrera 4 No. 20-41, Bogotá D.C. 110311, Colombia; Universidad Central, Carrera 5 No. 21-38, Bogotá D.C.110311, Colombia

**Keywords:** synthetic biology, education, exchange, non-monetary benefit-sharing

## Abstract

Synthetic biology and the emerging field of biomanufacturing provide new research concepts that should be incorporated into the training of the next generation of scientists. Key elements of synthetic biology are the standardization, modularization, and characterization of biological parts. Defined parts can be assembled into functional transcription units to pathways of multiple units, chromosomes, and/or whole genomes. Here, we highlight the importance of global exchange and state-of-the-art training in the Global South to empower research and reduce the gap between countries. Based on initial seed funding, we established a multidisciplinary bilateral exchange program between Germany and Colombia, including lecture series, experimental workshops, and bilateral exchanges in training and research. The elements are adaptable and our hands-on courses can be carried out with limited resources while covering aspects of synthetic biology. Importantly, this initiative will allow early-career researchers to build an international network, opening up opportunities in research collaboration and paving the way for greater equity.

## Graphical Abstract

**Graphical Abstract:**
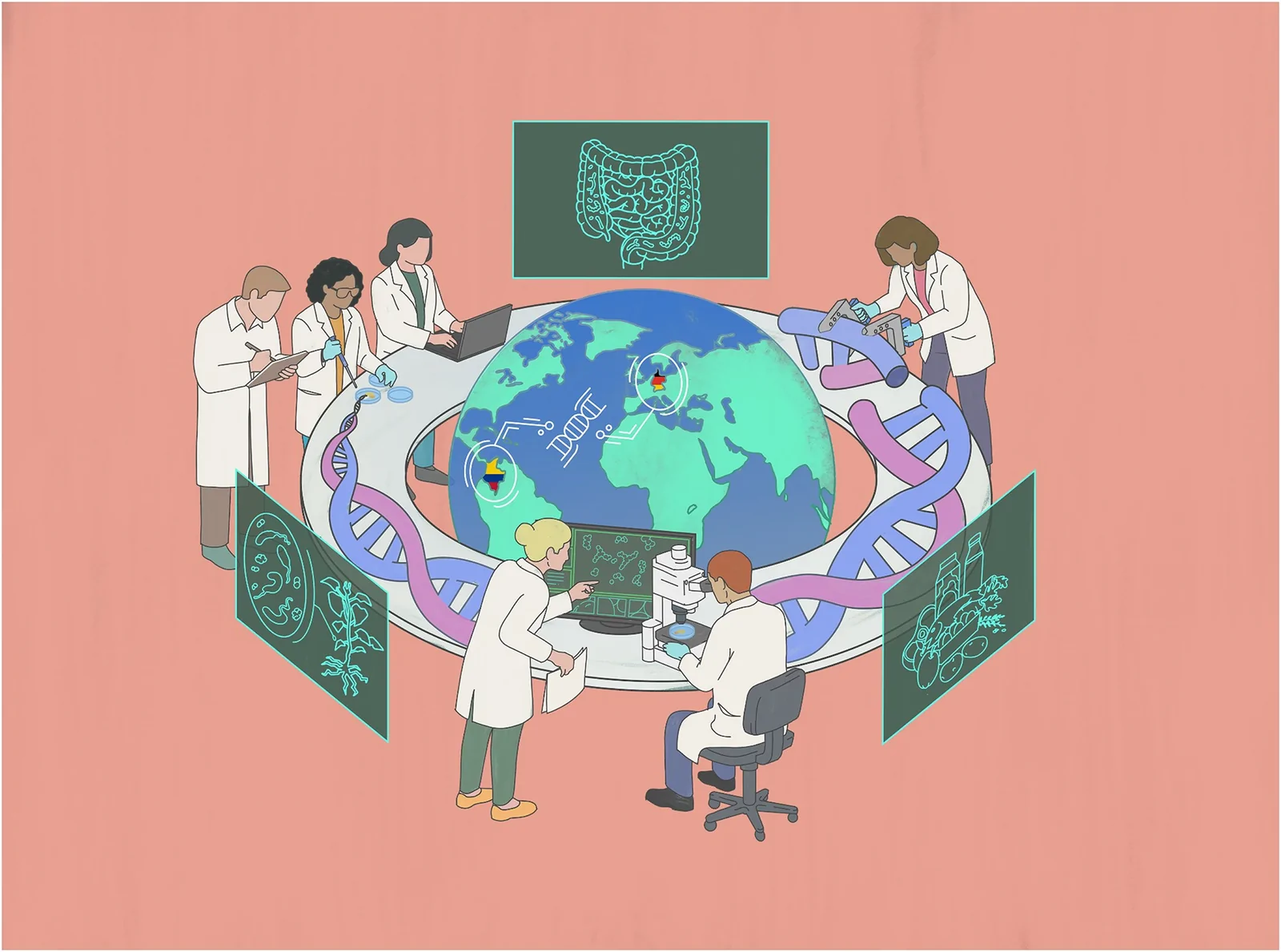

## Introduction

The field of synthetic biology emerged around the year 2000, and after >25 years, it is coming of age [1–4]. However, synthetic biology is still described as an emerging field. But what is synthetic biology? According to Wikipedia in 2026, it is “*a multidisciplinary field of science that focuses on living systems and organisms and applies engineering principles to design new biological parts, devices and systems, or to redesign existing systems found in nature* [5]”. This definition, which is intended for a broad audience, does not include aspects such as the use of synthetically produced building blocks (e.g. non-natural amino acids [6], synthesized DNA [7], and synthetic cells [8]). Synthetic biology also involves standardization and modularization to generate highly characterized component libraries, which can be used to assemble functional transcription unit modules [9–11]. This concept has revolutionized biology by accelerating DNA assembly through the integration of combinatorial approaches, standardized workflows, and laboratory automation. The result is highly automated facilities, so-called "biofoundries," which enable high-throughput approaches to engineering biology [12].

Synthetic biology has achieved important research milestones. One of these is the first redesigned and chemically synthesized *Escherichia coli* genome with a reduced set of codons, conferring resistance to viruses and allowing the use of non-canonical amino acids [13, 14]. Synthetic chromosomes and genomes may become more common as research into synthetic genomics accelerates [15]. This would unlock applications of synthetic organisms in biotechnology and biomanufacturing beyond metabolic engineering. One example is the use and incorporation into a circular bioeconomy of non-utilized resources and wastes [16].

However, the unequal distribution of capacities and facilities, such as large-scale infrastructures/centers for dedicated interdisciplinary applied and basic research found mostly in developed nations [12, 16], significantly hinders the widespread application of synthetic biology approaches. Nevertheless, synthetic biology can be carried out in settings with modest investment and infrastructure in laboratories equipped for molecular biology experiments—even in school classes [17]. Despite the availability of theoretical resources online [18], practical training is still a challenge. Greater engagement is required to facilitate understanding of concepts and elements required to set up an experimental design to address relevant research questions or applications. Targeted exchange and training programs can help close these gaps by providing the necessary background while introducing key synthetic biology concepts, research approaches, and practical applications.

This article provides insight into how we have carried out education in synthetic biology through a bilateral exchange program, the CaptureExpress project funded by the German *Federal Ministry of Education and Research* (BMBF) and the Colombian *Ministerio de Ciencia Tecnología e Innovación* (Minciencias). The exchange involved reciprocal visits that have opened opportunities at different levels, from introducing students to basic concepts in synthetic biology techniques and their applications to providing them the opportunity to apply for internships in cutting-edge research projects. This article presents the concept and the lessons learned from our multifaceted exchange. In the Supporting Information, we provide protocols for synthetic biology workshops that can be performed in standard molecular laboratory environments for experimental training in all aspects of the synthetic biology *Design-Build-Test-Learn* cycle (DBTL). This experience serves as a blueprint for other initiatives aimed at facilitating the sharing of knowledge and empowering the next generation of synthetic biologists (Fig. 1).

**Figure 1:**
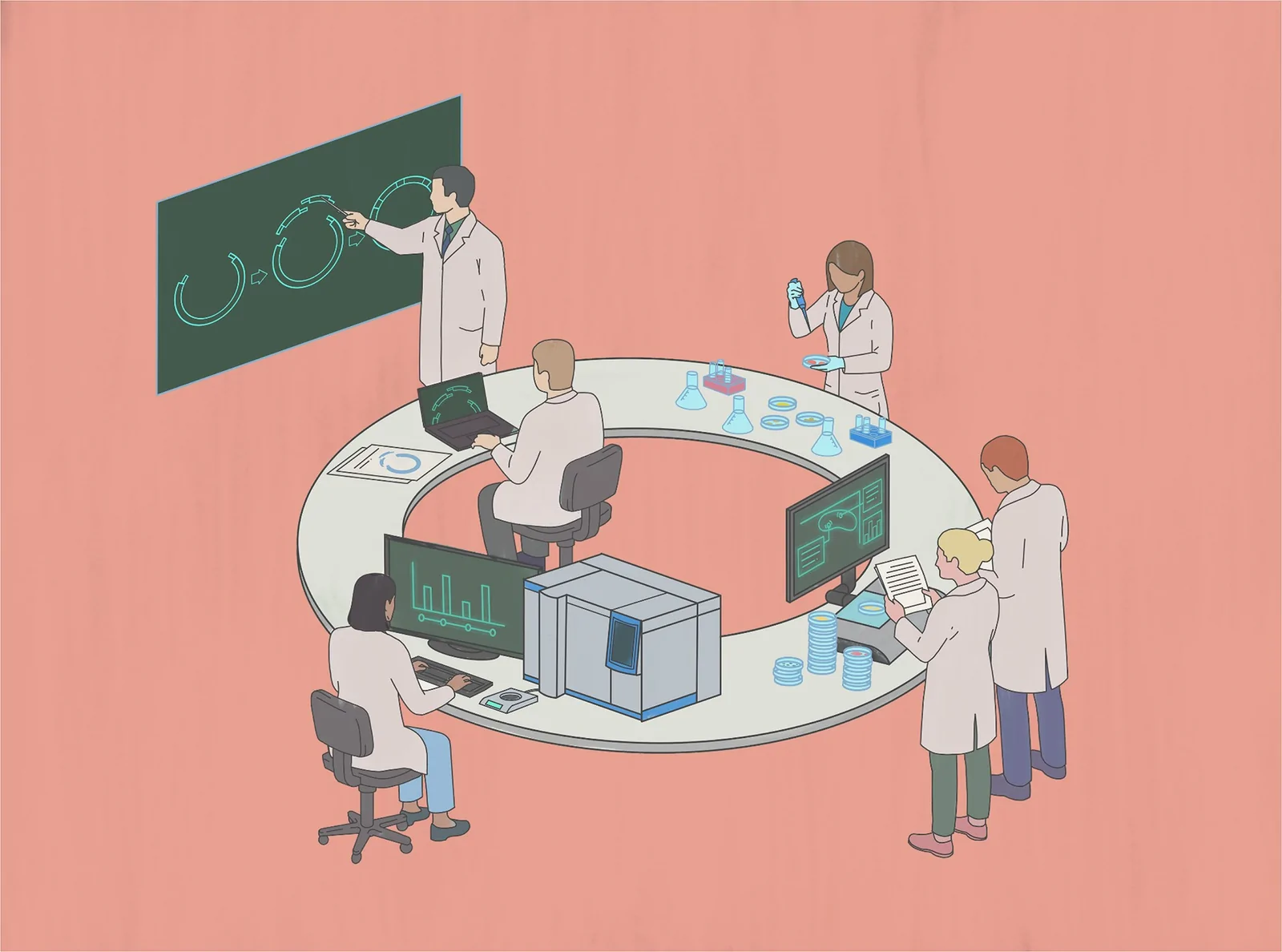
*Teaching synthetic biology using the DBTL cycle.* The core concepts of synthetic biology—standardization and logic engineering principles—can be taught with limited resources. Detailed protocols can be found in Supplementary Data 1 and 2.

## Program description

### Bilateral exchange to facilitate training and application of synthetic biology

Rectifying imbalances in the distribution of resources and access to research facilities can be achieved by generating opportunities for learning modern technologies and training in a competitive and international environment that fosters high research standards. The bilateral exchange of students and scientists benefits all parties involved: the establishment of research networks that facilitate the recruitment and training of talented individuals, the exchange of ideas, and access on both sides to materials and infrastructure. Training of early-career researchers fosters the development of future leaders, helps to bridge gaps, and contributes to non-monetary benefit sharing, a key consideration in current international discussions. The target groups can be divided into (i) undergraduate students, (ii) individual talents at different career stages, and (iii) diverse but topic-oriented groups.

#### Training for undergraduate students

Training for undergraduate students should consist of both theoretical and practical elements. In our exchange program, we used two different modules. The first was a lecture series consisting of two lectures per day for a week, delivered by local and guest researchers (Table 1). The seminars, which were intended to encourage extended informal discussions with the speakers, should preferably be open to the public and broadcast online. In our case, these lectures were held at Universidad Central in Bogotá in 2023 and 2024 and provided an overview of molecular biology and an introduction to various topics in synthetic biology (Table 1). A key feature was the inclusion of early-career researchers as speakers, who delivered seminars on their experiences of studying and/or doing a PhD abroad. The intention behind this was to highlight the benefits and challenges of studying abroad (e.g*.* cultural differences and establishing a social network) and blend personal and professional experiences. By doing so, they facilitated exchanges with interested students and provided a contact person who could serve as a role model or mentor and share first-hand experience. The seminar series was supported by experimental courses teaching the basics of molecular biology, such as polymerase chain reaction (PCR), molecular cloning, and plasmid transformation coupled to the observation of heterologous protein expression, such as fluorescence reporters. Depending on the available facilities, this may need to be restricted to a subset of interested students.

**Table 1:** Overview of exchange activities

**Event**	**Date**	**Participants**	**Venue**	**Modality**	**No. of speakers/instructors & affiliations**
Introduction to molecular and synthetic biology	Oct. 17–20, 2023	77 online and in-person	Universidad Central, Bogotá	Online and in-person lecture course	11; Max Planck-Marburg, U Central, U de los Andes, Corpogen
Basic synthetic biology workshop	Oct. 23–26, 2023	20 undergraduate students	Universidad Central, Bogotá	Workshop for undergraduates	3; Corpogen, Max Planck-Marburg
Advanced workshop -Golden Gate assembly	Oct. 17–20, 2023	10 Master’s and PhD students	Universidad de los Andes, Bogotá	Advanced workshop for graduate students	3; Max Planck-Marburg
Present and future of molecular and synthetic biology	Nov. 5–8, 2024	70 online and in-person participants	Universidad Central, Bogotá	Online and in-person lecture course	9; Max Planck-Marburg, U Central, U de los Andes, Harvard, Corpogen
Basic synthetic biology workshop	Oct. 22–25, 2024	20 undergraduate students	Universidad Central, Bogotá	Workshop for undergraduates	2; Corpogen, Max Planck-Marburg
Advanced workshop—Genome modification	Nov. 5–9, 2024	15 Master’s and PhD students	Universidad Central, Bogotá	Advanced workshop for graduate students	2; Max Planck-Marburg

Max Planck-Marburg, Max Planck Institute for Terrestrial Microbiology; U Central, Universidad Central, Bogotá; U de los Andes, Universidad de los Andes, Bogotá; Harvard, Harvard University.

#### Training for individual talents at different career stages

Training can be in advanced technologies and/or research-related topics. It should last multiple weeks to make the most of the hosting environment. Ideally, this is coupled with participation in a meeting or conference related to the research topic. Such an exchange benefits both partners and fosters bonding *via* joint research/training activities that facilitate future activities. If funds for mobility are limited, joint supervision of graduate-level projects is an excellent alternative, providing mentorship from two supervisors and enabling a research project to be approached from multiple angles. This is particularly valuable when students from both institutions are jointly investigating a research topic with frequent exchange through video conferences.

#### Training of diverse but topic-oriented groups

Advanced students and local researchers should carry out experimental and conceptual training tailored toward a specific goal, which must be carefully organized in advance to ensure all the logistics are in place. It is important to bear in mind that, in some countries, obtaining standard and custom consumables, such as enzymes and DNA oligonucleotides, can take weeks rather than days. Additionally, careful communication regarding the facilities and available equipment is important to ensure that experimental work can be conducted according to local legislation and to provide participants with the best possible experience.

Over the last few years, we have conducted multiple bilateral exchanges between Colombia and Germany. While (i) and (ii) can easily be tailored, (iii) is more complex. In the next section, we provide two examples of synthetic biology workshops that can be conducted in most molecular biology laboratories and that require standard materials and infrastructure (e.g*.* a PCR machine, a benchtop centrifuge and water baths). In Table 1 we provide an overview of the events and participants.

### Experimental teaching of synthetic biology with limited resources

A fundamental concept in synthetic biology is the adaptation of the DBTL from engineering to biology. This framework has, for example, been used to enhance specific biocatalysts, biosynthetic pathways, and production strains [19]. DBTL was integrated into the workshops to teach the principles of rational design for manufacturing DNA constructs and to test their functional applications (Fig. 1). We designed practical courses that can be conducted in standard molecular biology laboratories with minimal equipment requirements. They include all elements of the synthetic biology DBTL process, from the design to the construction and functional characterization of DNA constructs, as well as rapid producer strain optimization using synthetic yeast strains [20]. The first workshop focused on standardized DNA assembly methods in the form of Golden Gate cloning [21] by computationally designing [22] and experimentally building synthetic small regulatory RNAs [23, 24] to induce phenotypes in bacteria, such as susceptibility to oxacillin (a *β*-lactam antibiotic) and reduced motility, by altering protein abundances *in vivo* [25]. The second workshop covered *in vivo* DNA assembly in yeast, using the lycopene and violacein pathways as examples of assembly of multiple DNA fragments into functional plasmids *via* homologous recombination [26, 27]. Additionally, chassis engineering was covered using synthetic yeast strains that produce *β*-carotene and violacein at high yields [28]. Those yeast cells were SCRaMbLEd (SCRaMbLE = Synthetic Chromosome Rearrangement and Modification by LoxP-mediated Evolution [29]) to induce large-scale rearrangements in the synthetic chromosomes. The genomic alterations change the production of the molecules, which can be observed by eye (orange or dark pigmentation). Analysis of the obtained strains was completed by performing a mock Nanopore sequencing experiment and discussing the data analysis and interpretation. The trainees were particularly interested in the mock library preparation and handling/loading of Nanopore sequencing flow cells. Detailed protocols and DNA sequence information for both workshops are provided in the Supporting Information (Supplementary Data 1–3) and can be conducted in a standard molecular biology laboratory with basic molecular biology equipment.

### Fostering exchange opens opportunities

Our Colombia-Germany initiative started in 2023 and is still in its early stages. However, we realized that early-career researchers in particular would benefit from interactions with international researchers, which improve language skills and foster confidence. During the initiative, participants took part in programs to bolster their careers (Fig. 2), such as the 'Microcosm Earth Pre-doctoral Fellowship' program, an initiative from the Max Planck Institute for Terrestrial Microbiology and Philipps University Marburg. Promoting such programs and facilitating contact with researchers, including current early-career researchers, has increased the number of applications from highly talented individuals. In our case, two to three participants from our initiative were selected during the last years for the 6-month fellowship from a larger pool of applicants from the Global South. In 2025, two of them were subsequently accepted as PhD students at the International Max Planck Research School (IMPRS). Pre-doctoral fellowships, such as the 'Microcosm Earth Pre-doctoral Fellowship,' allow master's students to join a research group abroad for a period of time, while receiving a salary to cover associated expenses. In this environment, early-career researchers and potential PhD supervisors can interact and explore interest compatibility, increasing the likelihood of acceptance of students from the Global South. Such initiatives also promote an international and diverse environment where research flourishes from exchange and collaborative exploration. While the aforementioned programs are extremely valuable, they are not available everywhere. However, there are other ways to engage and promote early-career researchers, such as supervision of joint research projects that can be carried out remotely and may be complemented by a stay abroad. While students clearly benefit, these projects also promote interactions between researchers with the potential to collaborate on future projects.

**Figure 2:**
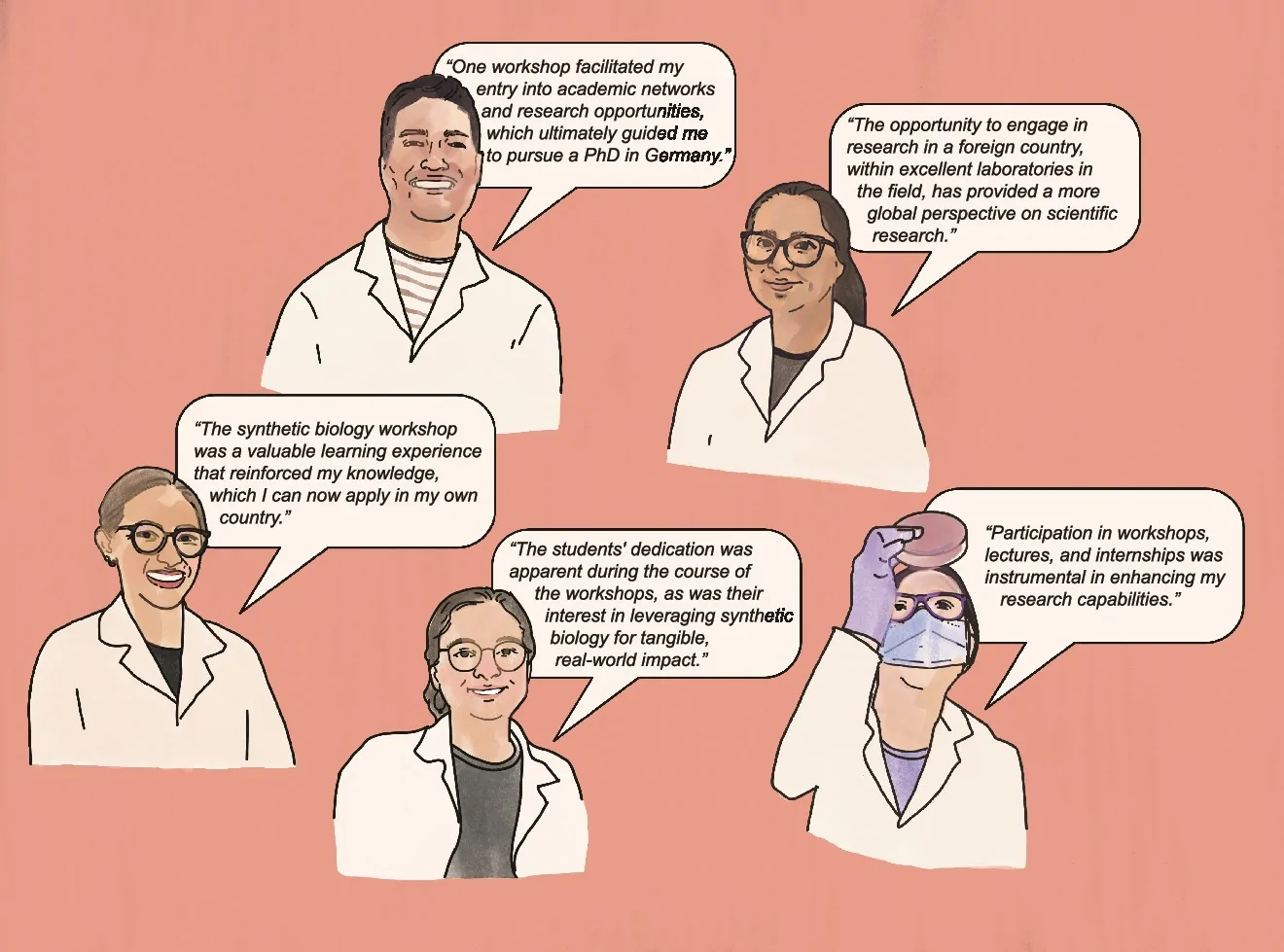
*Impression of initiative participants.* Quotes from students who participated in the German-Colombian exchange program, either as trainees or instructors. Notably, two of the participants were recruited as PhD students in Germany, building on the network they established during the program. Participants consented to their quotes being published in the context of this article, in a shortened and anonymized form, and agreed that the context is correct.

## Discussion

This article aims to encourage global exchange and non-monetary benefit sharing by creating opportunities for early-career researchers and promoting collaboration on joint projects and teaching. Training in current concepts and approaches, such as those used in synthetic biology, is important for keeping scientific education aligned with rapidly evolving research practices. To this end, in the Supporting Information we provide protocols for two experimental workshops that facilitate synthetic biology training even when resources are limited. Research thrives in diverse environments where new concepts and approaches enable innovative ideas and collaborations. While the initial start of such exchange programs may be bumpy, they rapidly develop their own dynamics, providing benefits for both sides by bridging gaps in training and generating opportunities for talented young scientists and collaborative work. It is rewarding to see the resulting track records of early-career researchers who participate in such initiatives, as they will become the future leaders in various positions. Those who have experienced international environments will promote cultural diversity in a world that is unfortunately shifting toward nationalism and protectionism.

### Material availability

All materials (plasmids and strains) are available from the corresponding authors upon request without an MTA. We encourage recipients to share these materials for training purposes afterwards. The synthetic yeast strains originate from the laboratory of Yizhi Cai (Manchester Institute of Biotechnology, UK) and are published and accessible *via* Liu *et al.* 2018 [28] and should be requested from the original source.

## Supplementary Material

Supplemtary_material_ysag012

## Data Availability

All data are available in the manuscript and its Supporting Information, or from the corresponding authors upon request.
