# Supplementary material for: Bilateral exchange in synthetic biology: experiences, challenges, and opportunities for training and research
Source: Synth Biol (Oxf). 2026 Oct 24;11(1):ysag012. doi: 10.1093/synbio/ysag012 (PMC13723260; doi:10.1093/synbio/ysag012)
Supplement: Supplementary file 1 [file supplemtary_material_ysag012.zip › Supplementary Data 1.pdf]

**Supporting Material I**

**Experimental workshop I**

# Bilateral exchange in synthetic biology: experiences, challenges, and opportunities for training and research

**Authors:** María del Carmen Sánchez Olmos, Martha Lorena Medina, Adán Andrés Ramírez Rojas, Nohora Angélica Millán Aldana, Paula Manuela Siaucho Unriza, Nicolas Londoño Sanchez, Valentina Florez Tellez, Victor Hugo Rua Lopez, Patricia del Portillo, Maryam Chaib De Mares, María Francisca Villegas-Torres, Daniel Schindler, María Mercedes Zambrano

*Escherichia coli* is one of the most important model organisms used in biology. Because it is easy to grow and manipulate, scientists have developed many molecular tools for this bacterium. These tools allow researchers to design and build plasmids, create new strains, and test how proteins work inside living cells. These tools are also essential in many biotechnological applications.

The two protocols in this module are designed to introduce students to essential laboratory techniques for introducing foreign DNA into *E. coli* by chemical transformation. Learning how *E. coli* takes up DNA is a foundational skill in molecular biology, as it is required for cloning, strain construction, and many types of genetic experiments and taught using the first protocol.

The second protocol expands on these skills by showing how small regulatory RNAs (sRNAs) can be used to control the expression of specific genes. This regulation produces measurable changes in phenotype, allowing students to observe how altering gene expression affects cell behavior. Finally, this protocol demonstrates how sRNAs can serve as powerful regulatory elements for studying gene function and gene essentiality. It also introduces students to core concepts used in synthetic biology, such as modular regulation and the design of synthetic parts

| Protocol no. | #1 |
|---|---|
| Title | *Escherichia coli* competent cell preparation with $CaCl_2$ |
| Keywords | **Competent *E. coli* cells** |
| Authors | Maria del Carmen Sánchez, Lorena Medina, Adán Andrés Ramírez Rojas, Maria Mercedes Zambrano, Daniel Schindler |


**Summary**

The preparation of *Escherichia coli* competent cells for DNA transformation through heat shock is a widely used molecular biology protocol. This protocol uses $CaCl_2$ to make *E. coli* cells chemically competent. DNA uptake by cells is facilitated by calcium ions' positive charge, which acts as a bridge between the phospholipids' negative charge in the cell membrane and the phosphate backbone of DNA (DeLucia *et al*., 2011; Liu *et al*., 2006). Transformed cells must then be selected using a marker cassette that either confers antibiotic resistance or uses a color marker such as GFP.


**Materials**

- Ice
- LB media
- 0.1 M $CaCl_2$ in $H_2O$
- Optional: 0.1 M $CaCl_2$ in 15% glycerol (for storage at -70 °C)

**Procedure**

| Preparation of competent *E. coli* cells<br>Step | Time | ☑ |
|---|---|---|
| Streak out *E. coli* in a Petri dish with the appropriate antibiotic from a glycerol stock | **Day 1** | ☐ |
| Incubate the strain at 37 °C overnight to obtain single colonies | | ☐ |
| Take a single colony from the Petri dish and grow in 5 mL of LB with the appropriate antibiotic. Incubate at 37 °C overnight with shaking (200 rpm) | **Day 2** | ☐ |
| From the overnight pre-culture prepare a main culture with 50 mL of LB with a final $OD_{600}$=0.01. Incubate at 37 °C with shaking (200 rpm) | **Day 3** | ☐ |
| Monitor the culture every hour until the $OD_{600}$ reaches a value between 0.3-0.5. Immediately put the cells on ice. | | ☐ |
| Harvest the cells at 10,000 × *g*, 4 °C for 5 min in a 50 mL falcon tube. Discard the supernatant carefully to not re-suspend the cells. | | ☐ |
| Re-suspend the cells carefully in 1 mL of cold 0.1 M $CaCl_2$. Incubate on ice 30 min. | | ☐ |
| Harvest the cells at 10,000 × *g*, 4 °C for 5 min. | | ☐ |
| Re-suspend the cells in 500 µL of cold 0.1M $CaCl_2$ | **~ 4 hours** | ☐ |
| **Note:** The $CaCl_2$ solution can be supplemented with 15% glycerol to be able to store the cells at -70 °C. Cells will be stable for one year. | | |

| Transformation of chemical-competent *E. coli* cells<br>**Step** | **Time** | ☑ |
|---|---|---|
| Use 50 µL of competent cells per transformation.<br>Transfer 50 µL of cells in a 1.5 mL sterile reaction tube. | | ☐ |
| Add 100 ng of DNA to the cells and incubate on ice for 30 min. | | ☐ |
| Set a water bath to 42 °C. Transfer samples to the water bath and incubate them for 30 seconds | | ☐ |
| Incubate on ice for 2 min. | | ☐ |
| Add 250 µL of pre-warm SOC or LB media to the reaction tube and incubate for 1 hour at 37 °C with shaking | | ☐ |
| Spread the cells with sterile glass beads or a cell spreader on appropriate selective media in a Petri dish. | | ☐ |
| Incubate the plates facing down overnight at 37 °C. | **~ 1:40 min** | ☐ |

**Note:** It is always recommended to have a negative control, without plasmid to test for potentialcontamination. In addition, it is important to determine the transformation efficiency by transforming defined amount of a known plasmid as positive control.

Formula to determine transformation efficiency (TE):

$$\frac{TE = (\#\, colonies/\ \mu g\ DNA)}{Dilution\ factor}$$

**Bibliography**

DeLucia, A. M., Six, D. A., Caughlan, R. E., Gee, P., Hunt, I., Lam, J. S., & Dean, C. R. (2011). Lipopolysaccharide (LPS) inner-core phosphates are required for complete LPS synthesis and transport to the outer membrane in *Pseudomonas aeruginosa* PAO1. mBio, 2(4), e00142-11.

Liu, I., Liu, M.M., & Shergill, K. (2006). The effect of spheroplast formation on the transformation efficiency in *Escherichia coli* DH 5 α.

| Protocol no. | #2 |
|---|---|
| Title | Synthetic sRNAs application: Susceptibility and swarming assays |
| Keywords | **Small RNAs, Golden Gate assembly** |
| Authors | Maria del Carmen Sánchez, Lorena Medina, Adán Andrés Ramírez Rojas, Maria Mercedes Zambrano, Daniel Schindler |

Summary

This part provides an introduction to synthetic biology through the cloning of synthetic regulatory RNAs that target AcrA mRNA a component of the AcrA-AcrB-TolC multidrug efflux pump and bacterial motility machinery by targeting FliC mRNA. Small regulatory RNAs (sRNAs) are prevalent in bacterial cells, where they use imperfect base pairing in their seed region with a set of target mRNA molecules to enable a rapid response to environmental changes. This process is known as post-transcriptional regulation, as it occurs at the mRNA level. It can increase or decrease the translation of target mRNAs. In this course, the *E. coli* RybB sRNA will be used as a blueprint for targeting *acrA* and *fliC* mRNA in order to reduce their respective protein abundances. RybB is a simple sRNA with a 5′ seed region of 16 nucleotides and a stem-loop structure that forms the sRNA scaffold (see Köbel *et al*., 2022, Brück *et al*., 2024). In this experimental setup, the RybB seed region is replaced with *acrA*- and *fliC* mRNA-specific target sequences predicted by computational analysis and inserted using Golden Gate cloning. *E. coli* cells are then transformed and the corresponding phenotype observed. Reducing AcrA makes *E. coli* cells susceptible to the β-lactam antibiotic oxacillin. Reducing FliC results in reduced motility of *E. coli* cells. These two phenotypes can easily be monitored and serve as an example to illustrate the modular cloning approaches and standardisation of core elements that are fundamental to synthetic biology.

**Materials**

- $ddH_2O$
- 10 x ligase buffer
- Type IIS enzyme (SapI)
- T4 ligase
- Basic part plasmid with promoter (pSL135; size: 2120 bps; GenBank files in Supporting Data)
- Seed-regions DNA (annealed oligonucleotides*)
    - i. *acrA*#1
    - ii. *acrA*#2
    - iii. *acrA*#3
    - iv. *fliC*#1
    - v. *fliC*#2
    - vi. *fliC*#3
  - ⇨ The forward and reverse oligonucleotide are annealed by boiling in a PCR reaction tube and slowly cooling down to room temperature to generate double stranded DNA.
- Basic part plasmid with sRNA scaffold (pSL123; size: 2228 bps; GenBank files in Supporting Data)
- Acceptor plasmid (pSL137; size: 4488 bps; GenBank files in Supporting Data)
- Respective LB and TB media, the latter for the swarming assay, with corresponding antibiotics

* This can be a computational design exercise using the SEEDling computational tool (Philipp et al., 2023), which allows to perform design on demand and get familiarised with containerised software (Docker; https://www.docker.com/).

**Procedure**

**sRNAs protocol: Susceptibility and swarming assays**

| Step | Time | ☑ |
|---|---|---|
| Before starting, check that you have all the materials required for this protocol | **Day 1** | ☐ |
| 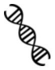 Prepare your Golden Gate reaction in a PCR tube (keep all on ice) | | ☐ |

| Reagent | Reaction | Concentration | Volume |
|---|---|---|---|
| Promoter DNA | 40 fmol | | |
| Seed-region DNA | 40 fmol | | |
| Scaffold DNA | 40 fmol | | |
| Acceptor plasmid | 40 fmol | | |
| 10 x ligase buffer | 1 x | 10 X | |
| Type IIS enzyme | 1 µL | NA | 1 µL |
| T4 ligase | 1 µL | NA | 1 µL |
| $ddH_2O$ | Add to total volume | NA | |
| Total volume | 20 µL | NA | 20 µL |

**Note:** as a general rule of thumb 80 ng of a 3 kb plasmid is equivalent to 40 fmol

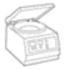 Transfer all reactions to a PCR cycler and use the following program: ☐

[4 min 37 °C - 4 min 16 °C]$_{25\ cycles}$; 10 min 50 °C; 20 min 65 °C; hold at 8 °C

**Day 2** 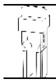 Perform transformation of 2 µL of Golden Gate reaction into *E. coli* cells (here *E. coli* MG1655 wild-type). Please refer to protocol #1 ☐

Prepare plates for the different assays: ☐

Antibiotic susceptibility – "*susceptibility plates*"

a. One plate with 50 µg/mL kanamycin (Km) and 0 µg/mL oxacillin
b. One plate with 50 µg/mL Km and 50 µg/mL oxacillin
c. One plate with 50 µg/mL Km and 100 µg/mL oxacillin

Bacterial motility – "*swarming plates*"

d. One plate for each strain with 50 µg/mL of Km and 1.5 % agar

Store plates carefully outside of direct sunlight or airflow to prevent evaporation.

**Day 3** Count the number of colonies per transformation and make an overnight in media ☐

**Day 4** **Swarming assay:** ☐

a. Take "swarming plates" and label plates properly according to the experiment. Do not label the lid!
b. Transfer 5 µL overnight culture on the center of the plate
c. Place control strain on each plate on the side in the same manner
d. Carefully move plate to a place for overnight incubation which is max. 30 °C and free of direct sunlight and constant air flow (e.g., air condition).

| | | |
|---|---|---|
| **Susceptibility assay:**<br>a. Take “susceptibility plates” and label plates properly. Draw a 6 x 3 grid on the plate to plate the dilutions<br>b. Generate 1:100 and 1:10,000 dilutions of each culture in a separate tube<br>c. Transfer 5 µL of the culture and both dilutions on its designated spot in the 6 x 3 grid.<br>d. Wait until remaining liquid evaporated<br>e. Transfer plates inverted into incubator and leave overnight at 37 °C | | ☐ |
| **Discussion and documentation of the results**<br>-Document your assays plates by taking pictures<br>-Perform qualitative analysis of swarming and susceptibility assay<br>-Document the diameter of the swarming assays using a ruler and calculate relative reduction of motility as a quantitative result. | **Day 5** | ☐ |

**Bibliography**


**Golden Gate cloning**

Engler, C., Kandzia, R., & Marillonnet, S. (2008). A one pot, one step, precision cloning method with high throughput capability. *PloS One*. 3(11), e3647.

Köbel, T. S., & Schindler, D. (2024). Automation and miniaturization of Golden Gate DNA assembly reactions using acoustic dispensers. Methods Mol Biol. 2850:149-169.

**Synthetic sRNA design**

Philipp, N., Brinkmann, C. K., Georg, J., Schindler, D., & Berghoff, B. A. (2023). DIGGER-Bac: prediction of seed regions for high-fidelity construction of synthetic small RNAs in bacteria. *Bioinformatics*. 39(5), btad285.

**Synthetic sRNA cloning**

Brück, M., Berghoff, B. A., & Schindler, D. (2024). *In silico* design, *in vitro* construction, and *in vivo* application of synthetic small regulatory RNAs in bacteria. Methods Mol Biol. 2760:479-507

Köbel, T. S., Melo Palhares, R., Fromm, C., Szymanski, W., Angelidou, G., Glatter, T., Georg, J., Berghoff, B. A., & Schindler, D. (2022). An easy-to-use plasmid toolset for efficient generation and benchmarking of synthetic small RNAs in bacteria. *ACS Synth Biol*. 11(9), 2989-3003.
