# Supplementary material for: Bilateral exchange in synthetic biology: experiences, challenges, and opportunities for training and research
Source: Synth Biol (Oxf). 2026 Oct 24;11(1):ysag012. doi: 10.1093/synbio/ysag012 (PMC13723260; doi:10.1093/synbio/ysag012)
Supplement: Supplementary file 1 [file supplemtary_material_ysag012.zip › Supplementary Data 2.pdf]

## Supporting Material II

## Experimental workshop II

# Bilateral exchange in synthetic biology: experiences, challenges, and opportunities for training and research

**Authors:** María del Carmen Sánchez Olmos, Martha Lorena Medina, Adán Andrés Ramírez Rojas, Nohora Angélica Millán Aldana, Paula Manuela Siaucho Unriza, Nicolas Londoño Sanchez, Valentina Florez Tellez, Victor Hugo Rua Lopez, Patricia del Portillo, Maryam Chaib De Mares, María Francisca Villegas-Torres, Daniel Schindler, María Mercedes Zambrano

Within the field of Synthetic Biology, routine protocols that support the assembly of complete transcriptional units and even full metabolic pathways are particularly valuable. They enable researchers to test new biological functions, explore novel pathways, and develop innovative solutions for the biotechnology sector.

These protocols leverage the natural capability of *Saccharomyces cerevisiae* to efficiently recombine DNA fragments, making it possible to construct and evaluate entire metabolic pathways, such as those involved in carotenoid production, protocol #3.

In addition, protocol #4 utilizes the power of the synthetic yeast genome to induce random large-scale recombination events in synthetic yeast genome strains. In this protocol *β*-carotene and violacein strains were used to SCRaMbLE their genomes and identify candidates which are either higher or lower producer strains. This was also used to discuss Nanopore sequencing and perform hands on experience by loading old, used flow cells (details not provided in this protocol).

Together, these protocols provide students with a foundational understanding of molecular biology, especially the principles underlying the assembly of individual DNA elements, such as promoters, terminators, and coding sequences and highlight the potential of yeast as a platform for building complete metabolic pathways.

| Protocol no. | #3 |
|---|---|
| Title | Assembling plasmids by homologous recombination in *Saccharomyces cerevisiae* |
| Keywords | **Homologous recombination** |
| Authors | Maria del Carmen Sánchez, Lorena Medina, Maria Mercedes Zambrano, Daniel Schindler |


**Summary**

*Saccharomyces cerevisiae* is a widely used eukaryotic microorganism in molecular biology, one of the reasons is its highly efficient recombination machinery. This feature enables precise integration of DNA at specific loci in its genome, as well as efficient recombination of two or more DNA fragments to assemble plasmids. For this process to occur, the required homology arms must be at least 25 nucleotides in length. In this protocol, we exploit this powerful yeast feature to assemble the *β*-carotene pathway within yeast using electroporation – the same approach would work with any other transformation method, for example the standard lithium acetate chemical transformation method.


**Materials**

- PCR reagents (DNA polymerase, buffer, primer, dNTPs, DNA template)
- YPD media
- Ice
- Sorbitol 1M
- SC-HIS + 2% glucose agar plates

***In yeasto*** **DNA assembly protocol**

| Step | Time | ☑ |
|---|---|---|
| Prepare PCR reactions (details of the plasmid and primers generating the different fragments are visualized in Fig. S1 and the plasmid is provided in the Supporting Data) as follows to amplify the linear vector and the different fragments of the carotenoid pathway: | **Day 1** | ☐ |

| **Components for the PCR master mix** | **Volume** |
|---|---|
| Buffer 10 x | 25 µL |
| $MgCl_2$ 2 mM | 20 µL |
| Forward primer 10 µM | 10 µL |
| Reverse Primer 10 µM | 10 µL |
| dNTPs | 50 µL |
| DNA (~2 ng/µL) | 1 µL |
| Taq polymerase | 5 µL |
| $ddH_20$ | 110 µL |
| Total | 250 µL |

| Fragment name | Forward primer (5´→ 3´) | Reverse primer (5´→ 3´) | Template DNA | Amplicon size |
|---|---|---|---|---|
| Amplified backbone | ggccgggctactctctatgatac | ggcgtgaatgaactttaagcgttgg | pSL0891 | 3354 bp |
| Fragment A (*crtYB crtI* and *crtE*) | gtatttgttggcgatccccc | gcagtttcatttgatgctcgatgag | pSL0891 | 7931 bp |
| Fragment B (*crtYB* and *crtI* or *crtYB* ) | gtatttgttggcgatccccc | ttcgaatgatccagctgtcc | pSL0891 | 5747 bp |

| | | |
|---|---|---|
| Analyze amplified DNA via gel electrophoresis and perform DNA clean-up with the preferred method prior to transformation into yeast. | | |
| Overview of the required fragments to transform in yeast (Fig. S1):<br><br>Positive control: 100 ng of plasmid pSL0891 (sequence provided in Supporting Data).<br>Negative control: 100 ng of amplified backbone from the pSL0891 (without pathway).<br>Assembly 1: 300 ng fragment A and 100 ng linear amplified backbone of the plasmid. This should result in a positive assembly with orange yeast colonies.<br>Assembly 2: 300 ng fragment B and 100 ng linear amplified backbone of the plasmid. This should result in no colonies and serves as additional negative control. | | |
| **Yeast transformation: Electrocompetent cell preparation** | | |
| Inoculate an overnight pre-culture of a respective yeast strain e.g., BY4741 in YPD medium from a single colony. | | |
| Inoculate 100 mL of YPD medium to a starting $OD_{600}$ of 0.1 from the overnight pre-culture and incubate at 30°C until the culture reaches and $OD_{600}$ = 1.3 – 1.5 (approx. $1*10^8$ cells/mL). | **Day 2** | |
| Harvest the cells by centrifugation 4,000 × *g* for 5 min at 4 °C. Discard supernatant. | | |
| Wash the cells in 50 mL with ice-cold sterile water, repeat centrifugation. | | |
| Repeat the washing step with 25 mL of ice-cold water. | | |
| Resuspend the cells in 20 mL of cold 1M sorbitol and centrifuge as before. Discard supernatant. | | |
| Resuspend in 0.2 mL of 1 M of sorbitol and keep on ice. | **~ 5 hours** | |
| **Yeast transformation: Electroporation** | | |
| Aliquot 65 µL of cells in a pre-chilled sterile 1.5 mL tube and add the DNA Homogenize gently with the pipet several times. Incubate 5 min on ice. | | |
| Transfer the mixture into a prechilled 0.2 cm electroporation cuvette of the manufacturer of choice. | | |
| Wipe off the water from the cuvette and insert it into the device. Electroporate at voltage 1,500 V with an expected pulse time of around 5 ms. | | |
| Immediately after electroporation add 1 mL of 1 M sorbitol. | | |
| Carefully ransfer mixture into an 1.5 ml reaction tube using a pipette. | | |
| Spin down reaction mixture, discard supernatant and resuspend in 200 µL 1 M sorbitol. | | |

| | | |
|---|---|---|
| Plate the mixture on one selective plate (SC-His) and incubate at 30 °C for 48 hours. | **~ 20 min** | ☐ |
| After 48 hours count the number of colonies and compare white versus colored colonies to have an estimate about the correct plasmid assembly rate. | | |

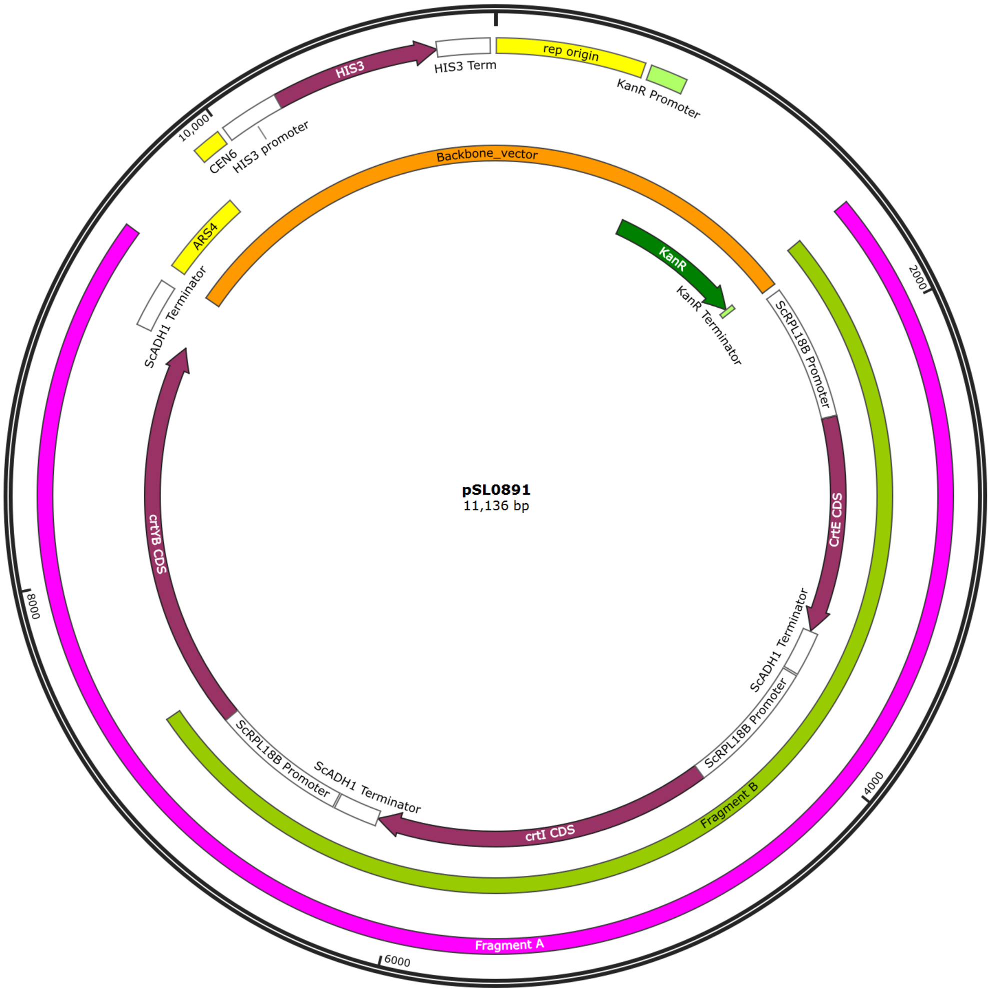


**Figure S1. Plasmid pSL0891 with the visualization of amplified segments.** Fragment A represents the whole pathway and fragment B only a partial fragment which serves as a control (Plasmid orignates from de Vries *et al*., 2024).

## Bibliography


de Vries, S. T., Köbel, T. S., Sanal, A., & Schindler, D. (2024). In- & Out-Cloning*:* Plasmid toolboxes for scarless transcription unit and modular Golden Gate acceptor plasmid assembly. *Synth Biol (Oxf)*. 7;9(1):ysae016.

| | |
|---|---|
| **Protocol no.** | #4 |
| **Title** | SCRaMbLEing of synthetic yeast |
| **Keywords** | Synthetic yeast |
| **Authors** | Maria del Carmen Sánchez, Lorena Medina, Maria Mercedes Zambrano, Daniel Schindler |

**Summary**

Big structural genomic rearrangements such as translocations, duplications, inversions and deletions happen at a very low frequency in yeast genome. However, these structural variations (SV's) are essential in the process of evolution. Thanks to the development of a system called SCRaMbLE (**S**ynthetic **C**hromosome **R**earrangement and **M**odification by **L**oxP-mediated **E**volution) within the synthetic yeast genome project (Sc.2.0) this process of large-scale genomic evolution can be induced and therefore sped up. SCRaMbLE relies on specific sequences called *loxPsym* sites inserted 3 bp after the stop codon of nearly all non-essential genes within the synthetic yeast chromosomes. When *β*-estradiol is added to the medium, the Cre-EBD can transfer to the nucleus and bind to *loxPsym* catalyzing SV's. The system has been successfully applied to evolve yeast cells to different conditions, for example it has been use to increase the tolerance to alkali by yeast (Ma *et al*., 2019). In this protocol we take a synthetic yeast strain producing *β*-carotene in high amounts as a proxy to estimate and identify SCRaMbLEd strains, as well as the ratio of induced/uninduced colony culture count to estimate the lethality rate after SCRaMbLE induction due to the loss of essential genes.

**Materials**

- SC-HIS + 2% glucose agar plates and liquid media
- *β*-estradiol
- 96-well microtiter plates
- YPD agar plates

| **Synthetic yeast SCRaMbLEing protocol** | | |
|---|---|---|
| **Step** | **Time** | ☑ |
| Streak out the synthetic yeast strains producing high amount of *β*-carotene. The strain used was LWy252 (Liu *et. al.,* 2018), transformed with the plasmid-pSCW11-CRE-EBD carrying the CRE recombinase (Lindstrom DL *et al.,* 2009). | **Day 1** | ☐ |
| Incubate the strain at 30 °C for two nights to obtain single colonies. | | ☐ |
| Isolate a single colony and prepare a pre-culture in 5 mL of SC-His. | **Day 3** | ☐ |
| Incubate overnight at 30 °C. | | ☐ |
| Measure the overnight pre-culture and inoculate two cultures per strain; grow to a final $OD_{600}$=0.1 | **Day 4** | ☐ |
| Add 1 µM of *β*-estradiol to one of the cultures for each strain to SCRaMbLE. Keep one tube without adding the inducer as a control. | | ☐ |

| | | |
|---|---|---|
| Take a sample of each tube after 2 hours, 4 hours and 6 hours. For each time point, make a dilution series in a 96 microtiter plate and spot every dilution on a SC-his plate and YPD plate. 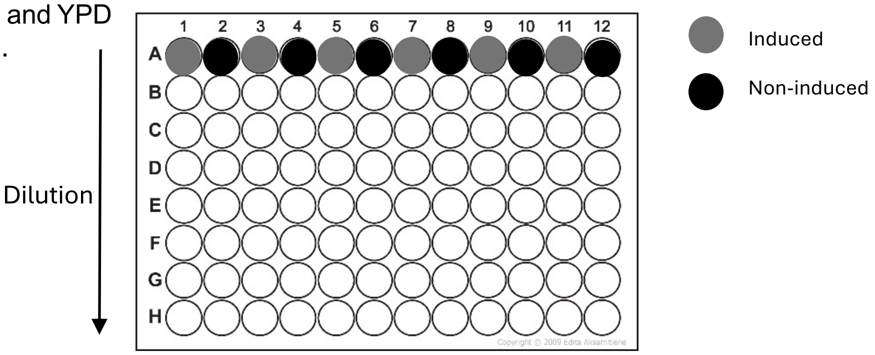  | **~ 6 hours** | ☐ |
| Incubate the plates at 30 °C for 48 hours and evaluate the obtained number of colonies. For the SCRaMbLEd strains you would expect a reduced viability and a potential loss or gain in the pathway activity. | | ☐ |
| **Note:** The sensitivity of each synthetic strain to Cre recombinase is quite different due to the presence of a different number of *loxPsym* and essential genes. The longer exposure to Cre recombinase will lead to not only more rearrangement events but also more cell death. The lethality can be measured by spotting assay at different time points on SC-His medium (Liu *et al.,* 2018)**.** | | |

**Bibliography**


Lindstrom, D. L., Gottschling, D. E. (2009) The mother enrichment program: a genetic system for facile replicative life span analysis in *Saccharomyces cerevisiae*. *Genetics.* 183:413-422.

Liu, W., Luo, Z., Wang, Y. *et al.*(2018). Rapid pathway prototyping and engineering using *in vitro* and *in vivo* synthetic genome SCRaMbLE-in methods. *Nat Commun* 9, 1936.

Ma, L., Li, Y., Chen, X., Ding, M., Wu, Y., & Yuan, Y.-J. (2019). SCRaMbLE generates evolved yeasts with increased alkali tolerance. *Microb Cell Fact.* 18(1), 52.
