# Supplementary material for: Bilateral exchange in synthetic biology: experiences, challenges, and opportunities for training and research
Source: Synth Biol (Oxf). 2026 Oct 24;11(1):ysag012. doi: 10.1093/synbio/ysag012 (PMC13723260; doi:10.1093/synbio/ysag012)
Supplement: Supplementary file 1 [file supplemtary_material_ysag012.zip › supplementary_material.docx]

Supporting Information

Supplementary Data 1 – Experimental protocol for workshop I (.pdf)

Supplementary Data 2 – Experimental protocol for workshop II (.pdf)

Supplementary Data 3 – GenBank sequences for all plasmids
